# Extending traditional antibody therapies: Novel discoveries in immunotherapy and clinical applications

**DOI:** 10.1016/j.omto.2021.08.005

**Published:** 2021-08-19

**Authors:** Charles Shin, Sung Soo Kim, Yong Hwa Jo

**Affiliations:** 1 Chadwick International, Incheon 22002, Republic of Korea; 2 Biomedical Science Institute, Kyung Hee University, Seoul 02447, Republic of Korea; 3 Department of Biochemistry and Molecular Biology, School of Medicine, Kyung Hee University, Seoul 02447, Republic of Korea

**Keywords:** therapeutic antibody, anti-cancer therapy, immune-related adverse effects, T lymphocytes, immunotherapy, cytotoxicity, apoptosis

## Abstract

Immunotherapy has been well regarded as one of the safer and antigen-specific anti-cancer treatments compared to first-generation chemotherapy. Since Coley’s discovery, researchers focused on engineering novel antibody-based therapies. Including artificial and modified antibodies, such as antibody fragments, antibody-drug conjugates, and synthetic mimetics, the variety of immunotherapy has been rapidly expanding in the last few decades. Genetic and chemical modifications to monoclonal antibody have been brought into academia, *in vivo* trials, and clinical applications. Here, we have looked around antibodies overall. First, we elucidate the antibody structure and its cytotoxicity mechanisms. Second, types of therapeutic antibodies are presented. Additionally, there is a summarized list of US Food and Drug Administration (FDA)-approved therapeutic antibodies and recent clinical trials. This review provides a comprehensive overview of both the general function of therapeutic antibodies and a few main variations in development, including recent advent with the proposed mechanism of actions, and we introduce types of therapeutic antibodies, clinical trials, and approved commercial immunotherapeutic drugs.

## Graphical abstract

**Figure:**
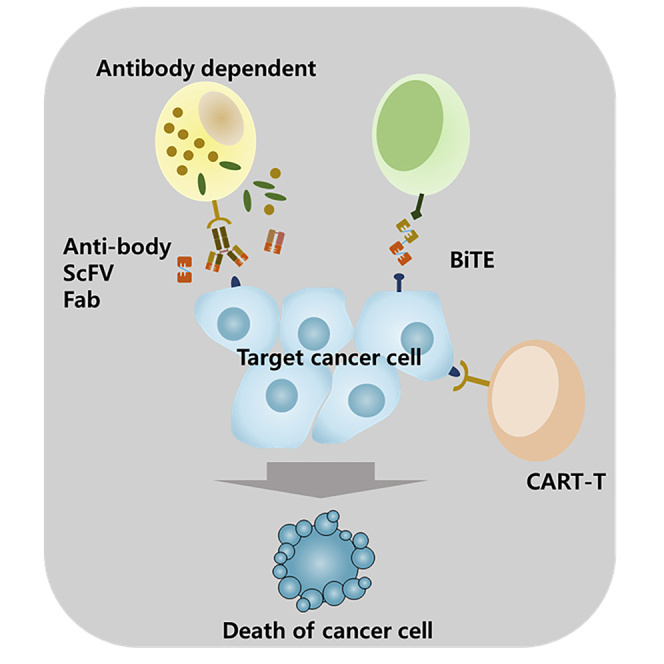

## Teaser

We provide a general overview of both clinical and experimental models of novel, modified antibodies along with their test case results. Overall, antibody extensions are coming close to becoming equal or more therapeutically favorable than traditional ones, especially in terms of bio-efficacy and toxicity.

## Introduction

Cancers, one of the most common and deadly diseases in human history, are notorious for their adaptiveness and persistence for survival. With over ten thousand genomic mutations possible, a single tumor cell is capable of controlling expression levels of specific proteins or tumor-associated antigens to optimize its environment.^1^ Acidic cancer microenvironment, one of the hallmarks for solid tumors, regulates cell-cell communication within and generates tumor-associated immune cells such as macrophages, regulatory T cells, and other leukocytes that produce immunosuppressive cytokines and chemokines to enhance tumor metastasis and proliferation.^2^^,^^3^ Such multi-faceted adjustability helps tumor cells evade or resist against established cancer therapies such as chemotherapy and radiation.^4^

From there comes immunotherapy, one of the most established studies upon recruiting the immune system of the patient for treatment of malignant tumors. The history of this immuno-oncological technique started when William B. Coley injected live and inactivated streptococcal bacteria into cancer patients to elicit erysipelas and trigger the immune system, which consequently eliminated malignant tumor cells.^5^^,^^6^ Despite receiving early criticisms, this immunostimulatory mixture, also known as “Coley’s toxin,” introduced to traditional oncology the fundamental concept of immunotherapy. Since then, application of immunotherapy has progressed from simple immunostimulatory cytokines such as interferon alpha (IFN-α) and interleukin (IL)-12 to more sophisticated techniques including oncolytic viruses, adoptive cell therapy, and immune checkpoint inhibitors.^7^

Antibodies are one of the most common biomolecules applied in immunotherapy. Since the discovery of cytotoxic T lymphocyte-associated antigen-4 (CTLA-4) and programmed cell death protein 1 (PD-1) as immune checkpoints by James P. Allison and Tasuku Honjo, respectively, multiple immunoglobulins (Igs) and their modified extensions have been developed to optimize anti-tumor response with both immunological and non-immunological pathways.^8^^,^^9^ However, few studies provide a general overview of both traditional and novel mechanisms of action involved in recently developed antibodies. This review intends to explain the fundamental biological structure, functions, and applications of therapeutic antibodies into immunotherapy with some of the most recent clinical results provided.

## Structures and functions of therapeutic antibody

### Structure of therapeutic antibody

**Figure 1:**
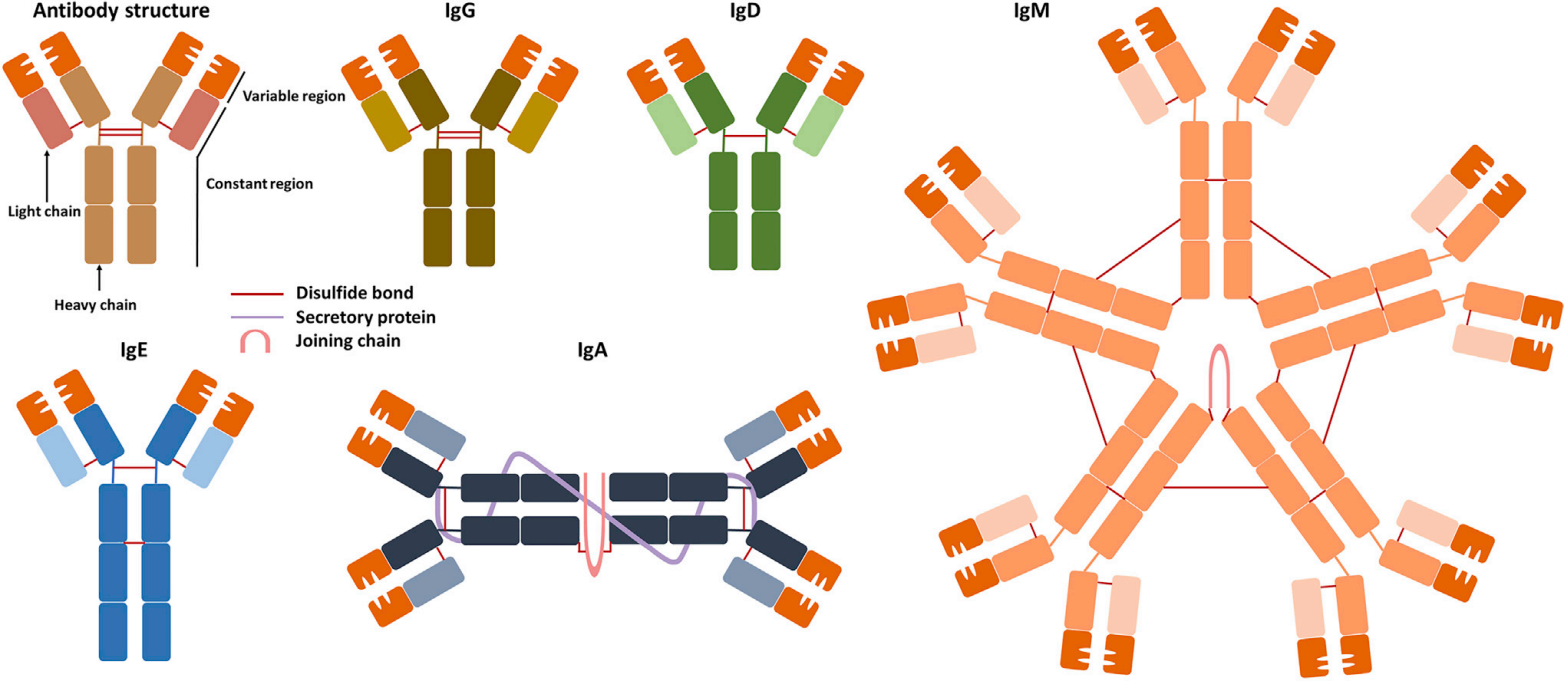
Antibody structures

Igs have five major classes where each class has a unique sequence of heavy chain (HC) constant regions: IgA, IgD, IgE, IgG, and IgM.^10^ IgG, which has a γ HC, comprises 80% of total antibodies in serum, making it most frequently used as immunomodulatory agents.^11^ Igs comprise of two HCs and light chains (LCs), each of which is further subdivided into variable domains and constant domains. The Y-shaped homodimer structure is linked by disulfide bonds nearby both the hinge region and the constant regions of LC (Figure 1).^10^

**Figure 2:**
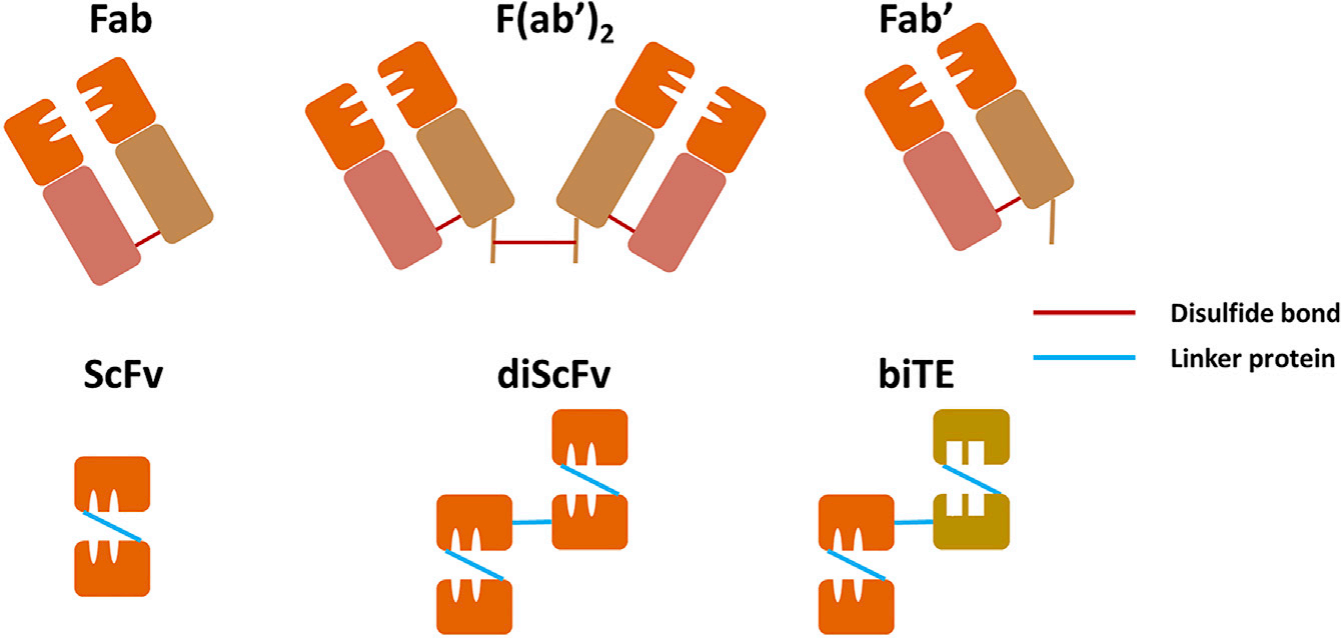
Fab and scFv structures

In functional units, Ig is divided into two antigen-binding fragments (Fabs), which bind to antigens by their paratopes, and one fragment crystallizable (Fc) region, which is responsible for the interaction between Ig and Fc receptors (FcRs).^12^ The variable regions of HC and LC can be isolated into a single-chain variable fragment (scFv), which is one of the smallest antigen-binding units for conventional antibodies. Each scFv is composed of two sets of three complementarity-determining regions (CDRs) and four framework regions (FRs).^13^ In humanizing antibodies from non-human species, the non-human CDRs are typically grafted into human FRs so as to preserve the paratope while lowering the immunogenicity.^14^ Various pharmaceutical industries have favored Fab and full-type humanizing antibodies. However, full-size antibody is difficult to be humanized repeatedly as it is usually developed using animal models. Modified antibodies can be synthesized at a faster rate than traditional, mouse-derived antibodies, because the former can be artificially synthesized with reference to an antibody library that contains humanized backbones (Figure 2).^15^

### Functional pathway of therapeutic antibody

Therapeutic antibodies bind to either soluble tumor-associated antigens or tumor cells directly so as to form an immune complex (IC).^16^ The Fc region can be used either as a interaction site for a complement system or as an opsonin for phagocytic cells such as macrophages and dendritic cells (DCs).^17^^,^^18^ These antibody-mediated immune effects mainly induce complement-dependent cytotoxicity, antibody-dependent cell-mediated cytotoxicity, and adaptive immunity due to antibody-dependent cell-mediated apoptosis.^19^ All three antibody functions cooperate to trigger both primary and secondary immune responses against tumor cells. Here, we discuss these three immune responses.

### Complement-dependent cytotoxicity

The complement system consists of over 30 glycoproteins. Most of them are mainly synthesized in the liver, although they can also be locally produced in various cell types such as macrophages, fibroblasts, and epithelial cells.^20^ Complement activation can be induced by three different pathways: classical activation, lectin activation, and alternative activation.^21^ All three of these mechanisms converge to C3, where cascading reactions continue to form membrane attack complex (MAC).^22^ The classical activation pathway is especially deeply related to antibody attachment on the membrane of target cells.

In classical activation, the C1 complex (C1qr2s2) forms C1q-Fc binding near the CH_2_-CH_2_ junction of IgG antibodies that are latched onto the membrane surface of a targeted tumor.^23^ Although unclear, the proposed model suggests that the C1 complex undergoes a conformational change induced by its interaction with IgG Fc, which activates serine protease C1r to cleave C1s in the tetramer.^24^ The activated C1s cleaves C4 into C4a and C4b, which then cleaves C2 to generate C2a. After C4b and C2a form the C3 convertase (C4bC2a) together, the fused complex cleaves C3 into C3a and C3b.^25^ From there, C3b covalently latches onto the outer leaflet of the cell membrane. After such reactions, soluble cleavage products (e.g., C3a, C4a) can induce stimulation of effector cells such as macrophages and downregulate expression of inhibitory fragment crystallizable gamma receptors (FcγRs), enhancing antibody-mediated cytolytic activities of effector cells.^19^

Multiple inhibitors exist both in normal and tumor cells to downregulate any uncontrolled complement activations. Factor H, factor I, C4BP, CD35, CD46, and CD55 have been discovered to effectively inhibit activities of C3 convertases.^26^ Normally, C3b2B recruits C5 to convert it into an alternative C5b convertase, which engages the C6−C8 complex and C9 in a cascading manner to form a perforating C5−C9 complex.^27^ However, factor I and factor H cut the C3b to iC3b for inhibition. Other negative regulators include clusterin, vitronectin, and CD59, which downregulate MAC formation by blocking C6−C9 complex formation.^28^ Several types of tumor cells, especially solid tumors, overexpress a few of these complement regulators such as CD46, CD55, and CD59, giving them resistance to therapeutic IgG antibodies *in vivo*.^29^ In fact, multiple bladder tumor cell types, including transitional cell carcinomas, adenocarcinomas, and squamous cell carcinoma, exhibited upregulation in membrane complement inhibitors (CD46: 77%, CD55: 55%, CD59: 59%) after the cell samples were incubated with anti-MUC1 (mucin-1) antibodies.^30^

### Antibody-dependent cell-mediated cytotoxicity

IgG antibodies also induce antibody-dependent cellular cytotoxicity (ADCC) with key effector cells such as natural killer (NK) cells, monocytes, and granulocytes so as to kill IgG-coated tumor cells.^31^ Type 1 FcγRs (FcγRI, FcγRIIa, FcγRIIc, FcγRIIIa, FcγRIIIb, FcγRIIb) mainly interact with IgG Fc, where all except FcγRIIb are activatory receptors.^32^^,^^33^ From this list, FcγRI (CD64), FcγRII (CD32), and especially FcγRIIIa (CD16) mediate IgG-dependent ADCC predominantly with NK cells—cell types that lyse cells that express MHC-I related chain (MIC)-A or MIC-B (indicators of stressed cells) or do not express major histocompatibility complex (MHC) class I complex.^34^^,^^35^ NK cells mediate cell death by releasing cytolytic granules, granzymes, perforins, and other cytotoxic effector molecules.^36^ This is because FcγRIIIa is only expressed on NK cells and macrophages, whereas FcγRI and FcγRII are widely expressed in multiple effector cells such as macrophages, neutrophils, eosinophils, and DCs.^37^ Furthermore, NK cells express activating FcγR only (CD16a, CD32c), making the cell type the key effectors of ADCC.^36^

The current ADCC signaling model is limited to FcγRIIIa-positive NK cells, but it thoroughly explains the cascading reactions necessary for targeted cell death. After IgG Fc binds to FcγR, the immunoreceptor tyrosine-based activation motif (ITAM) from the γ subunit is phosphorylated by SRC proto-oncogene, non-receptor tyrosine (src) kinases that are activated due to the crosslinking signal.^38^ Spleen tyrosine kinases (syks) are then recruited to phosphorylated ITAMs, by which the kinases are activated by their Src homology 2 (SH2) domains. Activating syk proteins induce the phospholipase C-gamma pathway (PLC-γ), phosphatidylinositol 3-kinase (PI3K) pathway, and Vav-Rho-family G-proteins pathway.^39^ In the PLC-γ pathway, syk proteins phosphorylate PLC-γ isozymes, triggering them to cleave phosphatidylinositol 4,5-bisphosphate (PIP_2_) to create inositol-1,4,5-triphosphate (IP_3_) and diacylglycerol (DAG).^40^ IP_3_ increases the intracellular calcium concentration, which is necessary to mobilize and discharge cytotoxic granules to targeted tumor cells.^41^ In the PI3K pathway, syk proteins activates PI3K proteins, subsequently polarizing the membrane surface with signaling proteins and enhancing signal transduction pathways.^42^ Finally, in the Vav-Rho-family G-proteins pathway, phosphorylated Vav proteins converge granules to a microtubule organizing center (MTOC), inducing granule exocytosis for cell death.^43^^,^^44^

### Adaptive immunity: Cross-over between Igs and immune cells

Therapeutic IgG can induce adaptive immunity in T cells by inducing phagocytosis and presentation of tumor-associated antigens from DCs. The antibodies trigger cellular phagocytosis by coating on tumor-associated antigens or tumor cells via paratope-epitope binding. Not only does creating ICs neutralize tumor-associated antigens, but this process also induces opsonization by altering the structural conformation of Fc so as to have a high affinity to FcγR, a family of FcRs that antigen-presenting cells (APCs) such as macrophages use to engulf bound components and degrade them in phagolysosomes.^45^ Glycosylated regions of Fc regions, primarily the N-linked glycosylation site in asparagine 297, play key roles in increasing the exposure of Fc regions around IC or antibody-coated cells to FcγR; the open conformation also increases Fc affinity to neonatal Fc receptor (FcRn) and regulatory lectins.^46^ In fact, Fc sialylation was discovered to directly modulate a humoral immune response of HIV gp120-specific antibody (PGT121) against HIV.^47^ The affinity of FcγRs also depends on IgG isotypes. During such internalization, actin-dependent cytoskeletal arrangements consisting of PI3K, Rac1, Cdc42, and myosin facilitate uptake of large IC particles.^37^ Antibody-mediated phagocytosis gives different outcomes depending on the type of APC; macrophages enhance pathogen destruction and antigen presentation, whereas plasmacytoid DCs enhance secretion of IFN-α to stimulate T cells and other immune cells against the tumor.^32^ Elotuzumab, a humanized IgG1 monoclonal antibody (mAb) approved by the US Food and Drug Administration (FDA) in 2014 to be used with dexamethasone and lenalidomide against relapsed or refractory multiple myeloma (MM), has been recently discovered to depend on antibody-dependent cellular phagocytosis with macrophages over NK cellular activation by signaling lymphocytic activation molecule (SLAM)F7 interaction for high anti-tumor efficacy.^48^

Cross-linkage with FcγRs activates various immune cells and is crucial to co-stimulate T cells through CD28-CD80/CD86 interaction and cytokine release. FcγR-IC aggregates then undergo clathrin-mediated endocytosis to subsequently fuse with lysosomes for proteolytic degradation. With cathepsin and human leukocyte antigen DM (HLA-DM), the remaining 13−25 amino acid fragments of FcγR and IC are loaded onto MHC class II molecules. Once completing the process, DC displays MHC class II complexes from endolysosomal vesicles to the cell surface. IC complexes could also be processed similarly with endogenous proteins to be fragmentated into 9−10 amino acid lengths, which can then be shuttled into MHC class I molecules through cross-presentation. By displaying both MHC class I and II complexes, DCs can present peptides from apoptotic tumors and elicit responses from both CD4^+^ T helper cells as well as antigen-specific CD8^+^ T cells.^49^ Indeed, a study has successfully induced cytotoxic T lymphocytes specifically against an autologous breast tumor after co-incubating alpha-type 1-polarized DCs (αDC1s) with breast cancer cell line MCF-7 that has been irradiated with ultraviolet light.^50^

However, ICs could also bind to FcγRIIIb, inducing cell deactivation through the immunoreceptor tyrosine-based inhibition motif (ITIM) pathway: SH2 containing inositol 5′ polyphosphatase (SHIP) dephosphorylates activating receptors and signal intermediates such as syks and PIP_2_, respectively.^51^ Nevertheless, internalizing ICs has been proven to enhance cross-presentation compared to uptaking soluble, non-IgG-bound antigens.^52^ Furthermore, against tumors that do not express MHC class II or have mutated defects, APC uptake of tumor-associated antigens is crucial for an effective CD4^+^ T helper response.^53^

### Extensions in therapeutic antibody

With advancements in molecular biology and genetic engineering techniques, therapeutic antibodies are fragmentated to heighten intratumor penetration capacity and to be able to bind specific epitopes that full-size antibodies cannot do, due to their large size. Some of the commonly recognized antibody fragments include scFv, nanobodies, and Fab. Herein, we will show various forms of therapeutic antibodies along with their clinical uses (Table 1).

**Table 1:** FDA-approved monoclonal antibodies (mAbs) for cancer

mAb	Target	Format	Indicant
Alemtuzumab	CD52	humanized IgG1	chronic myeloid leukemia
Atezolizumab	PD-L1	humanized IgG1	bladder cancer
Avelumab	PD-L1	human IgG1	Merkel cell carcinoma
Belantamab mafodotin	BCMA	humanized IgG1; ADC	refractory multiple myeloma
Bevacizumab	VEGF-A	humanized IgG1	colorectal cancer
Blinatumomab	CD19, CD3	murine bi-specific tandem scFv	acute lymphoblastic leukemia
Brentuximab vedotin	CD30	chimeric IgG1; ADC	Hodgkin lymphoma
			systemic anaplastic
			large cell lymphoma
Brolucizumab	VEGF-A	humanized scFv	neovascular age-related macular degeneration
Caplacizumab	VWF	humanized nanobody	acquired thrombotic thrombocytopenic purpura
Cemiplimab	PD-1	human mAb	cutaneous squamous cell carcinoma
Certolizumab	TNF-α	humanized Fab	Crohn’s disease
Cetuximab	EGFR	chimeric IgG1	colorectal cancer
Daratumumab	CD38	human IgG1	multiple myeloma
Durvalumab	PD-L1	human IgG1	bladder cancer
Elotuzumab	SLAMF7	humanized IgG1	multiple myeloma
Enfortumab vedotin	Nectin-4	humanized IgG1; ADC	bladder cancer
Gemtuzumab ozogamicin	CD33	humanized IgG4; ADC	acute myeloid leukemia
Ibritumomab tiuxetan	CD20	murine IgG1	non-Hodgkin lymphoma
Inotuzumab ozogamicin	CD22	humanized IgG4	acute lymphoblastic leukemia
Ipilimumab	CTLA-4	human IgG1	metastatic melanoma
Moxetumomab pasudotox	CD22	murine IgG1 dsFv	hairy cell leukemia
Necitumumab	EGFR	human IgG1	non-small cell lung cancer
Nivolumab	PD-1	human IgG4	melanoma, non-small cell lung cancer
Obinutuzumab	CD20	humanized, glycoengineered IgG1	chronic lymphocytic leukemia
Oportuzumab monatox	EpCAM	humanized scFv; ADC	bladder cancer
Panitumumab	EGFR	human IgG2	colorectal cancer
Pembrolizumab	PD-1	humanized IgG4	melanoma
Pertuzumab	HER2	humanized IgG1	breast cancer
Ramucirumab	VEGFR2	human IgG1	gastric cancer
Ranibizumab	VEGF-A	humanized Fab	macular edema
Rituximab	CD20	chimeric IgG1	non-Hodgkin lymphoma
Sacituzumab govitecan	Trop-2	humanized IgG1; ADC	breast cancer
Trastuzumab	HER2	humanized IgG1	breast cancer
Trastuzumab emtansine	HER2	humanized IgG1; ADC	breast cancer

ADC, antibody drug conjugate; CD, cluster of differentiation; dsFv, double-strand variable fragment; PD-L1, programmed death ligand 1; BCMA, B cell mutation antigen; VEGF, vascular endothelial growth factor; VWF, von Willebrand factor; PD-1, programmed cell death protein-1; TNF-α, tumor necrosis factor α; EGFR, epithelial growth factor receptor; SLAM, signaling lymphocytic activation molecule; CTLA-4, cytotoxic T-lymphocyte-associated protein 4; EpCAM, epithelial cellular adhesion molecule; Trop-2, tumor-associated calcium signal transducer 2; HER2, human epidermal growth factor receptor 2.

### scFv

scFv consists of a HC variable region connected to a LC variable region by a flexible linker, which is largely composed of serine and glycine amino acids.^54^ scFv has a short elimination half-life (5 h) caused by the low molecular weight (25 kDa) and no interaction of FcRn receptors during renal clearance.^55^ Diabodies, tribodies, tetrabodies, and other forms of multimers were constructed to raise the PK properties and binding affinities of scFv by reducing linker residue lengths.^56^

Several scFvs were approved for clinical use. Brolucizumab, a humanized anti-vascular endothelial growth factor A (VEGF-A) scFv to treat neovascular age-related macular degeneration (nAMD), has demonstrated a greater durability and effectiveness in a 12-week regimen compared to conventional treatments, which depended on directly injecting anti-VEGF-A solutions.^57^ For anti-cancer treatments, scFvs in various development stages are conjugated with immune stimulators, toxins, or antibody fragments (e.g., scFv, Fab, Fc) to increase *in vivo* retention rate or anti-neoplastic potency. Oportuzumab montax is a recombinant-fused protein linking humanized anti-epithelial cell adhesion molecule (EpCAM) scFv to truncated *Pseudomonas* exotoxin A. EpCAM is a homotypic cell adhesion glycoprotein that is often co-expressed as a complex with other receptors (e.g., CO-029, CD44v6, claudin-7) on both normal and cancerous epithelial cells.^58^^,^^59^ In assessing its effectiveness against bladder urothelial carcinoma that was resistant to bacillus Calmette-Guérin (BCG) treatment *in situ* in a phase II trial, 44% of patients got a complete response, and 16% continued to be disease free at the end of the study. Considerable bladder symptoms were displayed, but the therapeutic benefit outweighed the adverse effect. A phase III trial with BCG-resistant non-muscle invasive bladder cancer is ongoing right now.^60^^,^^61^ In 2017, Agha Amiri et al.^62^ developed anti-CD22 scFv-apoptin fusion protein to treat B cell malignancies. CD22 is a transmembrane glycoprotein that is specifically expressed on B cell surfaces for cellular function, survival, and apoptosis. Although the antigen is expressed in low numbers in normal B cells, 60%–80% of B cell lymphomas or leukemias such as non-Hodgkin’s lymphoma (NHL) express this antigen.^63^ With apoptosis-inducing protein (apoptin) recruited for tumor apoptosis, the fusion protein demonstrated cytotoxicity in Raji cells while not binding to CD22-negative Jurkat cells as demonstrated in 3-(4,5-dimethylthiazol-2-yl)-2,5-diphenyltetrazolium bromide (MTT) assay and annexin V/propidium iodide flow cytometry analysis.^62^

Fusion proteins consisting of scFv with antibody fragments are recently gaining more attention, although still less studied than scFv toxins. Du et al.^64^ synthesized a recombinant scFv-Fc-IL-2 fusion protein to target human epidermal growth factor 2 (HER2)-positive cancer. HER2 is a commonly overexpressed receptor responsible for cell migration and apoptotic resistance in breast, gastric, and ovarian carcinomas.^65^, ^66^, ^67^ *In vivo* trials showed the fusion protein to be capable of significantly delaying both HER2-positive non-small cell lung cancer and HER2-positive breast cancer with 1 mg/kg dose.^64^ Anti-epidermal growth factor receptor (EGFR) scFv tetramers were also constructed in 2016 for anti-neoplastic enhancement and extended blood retention time. Although scFv multimerization depends on both structural and environmental factors, the candidate drug was less costly while retaining a high inhibitory effect and long *in vivo* half-life.^68^ Although most of current scFv immunotherapies depend on fusing with separate biomaterials, constant attention to its development is highly recommended for its potential extensions.

### Nanobodies

Nanobodies, or single-domain antibodies, are derived from HC antibodies (90 kDa), which lack a LC and HC constant domain 1 (CH1 domain).^69^^,^^70^ HC antibodies are commonly found as components of the humoral immune system in *Camelidae* species including *C. dromarius*, *C. bactrianus*, and *Lama glama*.^71^ Sharks such as *Orectolobus maculates* and *Ginglymostoma cirratum* and ratfish also contain similar functional antibodies that lack LCs; these antibodies are commonly known as Ig new antigen receptor (IgNAR), a homodimer of two HC (5 constant domains each) and a single variable antigen-binding domain (variable new antigen receptor [VNAR]) each.^72^ Nanobodies are specifically the variable domains of camelid HC antibodies, or variable domain on a heavy chain (VHH). Having a molecular weight of 15 kDa, the VHH can penetrate endothelial barriers, blood-brain barriers, and plasma membranes with a high stability in both pH and temperatures.^73^^,^^74^ Since VHH was not linked to LC before purification, it is intrinsically hydrophilic, making it very soluble in non-mammalian expression systems too. VHH also has a sub-nanomolar affinity to antigens because it has an extended CDR3 loop structure that compensates for the absence of LC.^75^ Most importantly, camelid VHH is highly homologous to human heavy variable fragments by up to 80% of amino acid sequences.^76^^,^^77^ With these multiple advantages, some superior to conventional antibodies, nanobodies can bind to epitopes that were either not immunogenic or reachable for normal IgG molecules.

In 2019, the FDA approved an anti-von Willebrand factor nanobody, or caplacizumab, to treat acquired thrombotic thrombocytopenic purpura (aTTP). Caplacizumab targets the A1 domain of von Willebrand factor, inhibiting von Willebrand factor-platelet interactions.^78^^,^^79^ *In vitro* collagen perfusion studies have shown caplacizumab to completely inhibit adhesion of plasma platelets from patients undergoing percutaneous coronary intervention.^80^ More than one-half of patients daily treated with a 10-mg dose of caplacizumab have shown a significant reduction in time-to-platelet-count response compared with those with placebo. aTTP was also significantly reduced in those patients by nearly a 67% reduction (p < 0.001).^78^^,^^81^ For anti-cancer applications, a phase I trial was held where HER2-positive breast cancer patients were administered with ^131^I-labeled - 4-guanidino-methyl-3-iodobenzoate (^131^I-GMIB)-anti-HER2-VHH1 for targeted radionuclide theragnostics. Albeit the short plasma half-life (2.5 h) due to kidney filtration, the drug did not accumulate in thyroid and stomach. Adverse effects were absent, and sternal metastatic regions were clearly identified for 24 h after treatment in both single-photon emission computed tomography (SPECT) and computed tomography (CT) imaging.^82^ A similar phase I trial was conducted for ^68^Ga-1,4,7-triazacyclononane-1,4,7-triacetic acid (^68^Ga-NOTA)-anti-HER2 VHH1, which additionally underwent positron emission tomography (PET) imaging. Fast blood clearance was observed, and the tracer accumulation was substantial for identifying most sites of disease.^83^

Nanobodies that are not clinically trialed are also being actively developed for immunomodulation and other constructive functions. In 2012, Vosjan et al.^84^ developed an ^89^Zr-radiolabeled fusion protein composed of an anti-albumin nanobody (Alb8) and anti-hepatocyte growth factor (HGF) nanobody (1E2 or 6E10) for both anti-cancer toxicity and PET imaging *in vivo*; targeting albumin is generally accepted to extend an antibody’s half-life by pH-dependent, FcRn-mediated intracellular recycling. HGF, which is well known for its correlation to cancer aggressiveness and poor prognosis, is commonly overexpressed in solid tumors and acts as a single ligand for the MET proto-oncogene, receptor tyrosine kinase (c-Met) surface receptor.^85^^,^^86^ The anti-albumin system makes an improvement in the pharmacokinetic properties of otherwise short-lived molecules following conformational changes in structure at low pH or side-chain titration.^87^^,^^88^ Epithelial growth factor receptor nanobody with anti-albumin functionality refines tumor uptake and penetration while also extending elimination half-life.^89^ The fused nanobody complex has delayed tumor growth, showing promise in targeting soluble factors for immunotherapy like bevacizumab, an anti-VEGF antibody.^84^ Tumor-suppressive activities of recombinant nanobodies have been also reported along with lowering angiogenic biomarkers expressed on cancer cells. Khatibi et al.^90^ reported increased concentration of CD4^+^ and CD8^+^ tumor-infiltrating lymphocyte (TIL) in tumor microenvironment, dose-dependent retardation of tumor growth, and reduction in pro-angiogenic cytokines such as IL-1, IL-2, IL-12, and tumor necrosis factor α (TNF-α) by administering anti-CD3ε nanobodies (sequence provided by US 2011/0275787 A1) *in vivo*. Inhibiting the T cell receptor (TCR [CD3ε]) induced mitogenesis of T lymphocytes, demonstrating anti-angiogenic behavior that was aided with downregulation of VEGF receptor 2 (VEGFR2) and matrix metallopeptidase 9 (MMP9). Kaplan-Meier analysis also indicated a significant increase in survival rate for patients who received 3 μg/g anti-CD3ε nanobody compared to PBS control.^90^^,^^91^

### Multi-specific antibodies and bi-specific T cell engager (BiTE)

Multi-specific antibodies, especially bi-specific antibodies (bsAbs), are recombinantly produced to increase the number of recognizable antigens and the efficacy of redirecting immune effector cells to tumor cells.^92^^,^^93^ Not only does bsAb recruit immune cells for direct tumor destruction, but it also provides payload delivery and signal blockades. Production methods of bsAb includes quadroma, “knobs-into-holes,” chemical cross-linking, DuoBody, and CrossMAb.^55^^,^^94^ Full-length bsAbs are typically favored over traditional bivalent mono-specific antibodies because the former demonstrates greater Fc-mediated immune activities against tumor cells. The bsAbs are categorized into IgG-like antibodies and non-IgG-like antibodies, where the former is much more recognized than the latter due to their higher similarity to serum IgG molecules.^95^ Non-IgG-like bsAbs include BiTEs, tandem diabodies (TandAbs), and dual-affinity retargeting molecules (DARTs).^96^ These artificial macromolecules consist of antibody fragments (e.g., Fab, scFv, single-domain antibody [sdAb]) and linkers. BiTE will be further discussed later, whereas TandAbs and DARTs are thoroughly reviewed in other publications.^97^, ^98^, ^99^

Multi-specific antibodies have shown promises in tumor-specific destruction. In 2020, Kraman et al.^100^ reported that FS118, a bsAb targeting human lymphocyte activation gene-3 (LAG-3) and programmed death ligand 1 (PD-L1), enhances T cell activation and thus suppresses tumor growth. LAG-3 receptors are highly expressed on both human and mouse TILs; multiple studies have verified an enhancement of anti-tumor T cell responses in the presence of LAG-3.^101^ PD-L1 receptors are upregulated in multiple tumor cell types in response to proinflammatory cytokines (e.g., IFN-γ) so that they can inhibit activated TILs via PD-1/PD-L1 interaction.^102^ By blocking LAG-3 and PD-L1 simultaneously, FS118 can act as a bridge for TILs to perforate PD-L1-positive tumor cells.^100^ In 2016, Vallera et al.^103^ synthesized a novel 161533 tri-specific killer engager (TriKE) that is composed of anti-CD16 scFv, anti-CD33 scFv, and IL-15 so as to act as an immunologic linker between NK cells and CD33^+^ HL-60 myeloid cancer cells. Although binding to CD16 receptors on NK cells and CD33 receptors on tumor cells, TriKE can stimulate NK cell growth via IL-15. Through this mechanism, TriKE demonstrated far superiority in stimulating NK cell cytotoxicity, survival, and proliferation than previous bi-specific NK cell engagers.^103^ REGN1979, a humanized bi-specific anti-CD20 × anti-CD3 IgG4, recently completed an open-label, multi-centered phase I trial for improving survival rates of pre-treated patients who had relapsed/refractory advanced B cell NHL (B-NHL). With a reduced Fc binding affinity, the bi-specific monotherapy crosslinks with CD20^+^ B cells and CD3^+^ T cells, migrating T cells for tumor killing. Although pyrexia, cytokine release syndrome (CRS), and chills commonly emerged in patients with B-NHL, dose-limiting toxicities were not experienced, and CRS severity was controlled with appropriate pre-medications.^104^

BiTE antibodies are recombinantly produced by linking two scFvs with a glycine-serine peptide linker. Similar to full-length bsAbs, BiTE connects tumor cells to TILs by paratope-receptor interactions. BiTE typically targets TIL CD3 receptors, which co-stimulate both CD4^+^ and CD8^+^ T cell types.^105^^,^^106^ The bi-specific engager can also fuse with another scFv to generate a trivalent/tri-specific antibody. Although having a short half-life due to absence of Fc region and low molecular weight (55 kDa), BiTE has picomolar binding affinity to tumor-associated antigens in return.^107^ Furthermore, it can induce polyclonal T cell activation and proliferation by assisting TCRs to interact with cancer antigens while co-stimulating the activation pathway by binding to CD3 receptors.^106^^,^^108^ Blinatumomab (anti-CD3, CD19), a BiTE approved by the FDA in 2014 for treatment of relapsed and refractory precursor B cell acute lymphoid leukemia (ALL), was placed in a randomized phase III trial with chemotherapeutic agents to demonstrate its *in vivo* efficacy and tolerability against CD19-positive ALL in 2019. Brown et al.^109^ concluded that the immunologic agent exhibited less severe cytotoxicity, higher sensitivity to minimal residual disease, and a greater chance of an improved survival after treatment. Both IgG-like and non-IgG-like bsAbs have clinically demonstrated safer and more specific anti-tumor therapy compared to traditional medications. Overall, blinatumomab increased the relapse-free survival and achieves a molecular remission in B cell lymphoma.^110^ AMG420, a BiTE against B cell maturation antigen (BCMA) and CD3, completed a dose escalation, multi-centered phase I trial in 2019 where MM was treated by proximity-based T cell-mediated lysis of BCMA-positive cells such as MM cells and plasma cells. Although 48% (n = 20) of 42 patients experienced severe adverse effects including infections and polyneuropathy, central nervous system toxicities over grade 3 were not exhibited. Response rate for maximum tolerated dose (400 mg/day) recorded 70%, and one-half of the patients gave a complete response without minimal residual disease indication.^111^ Despite *in vivo* toxicity, BiTE displays an acute anti-cancer response, making it a promising immunotherapeutic agent in the near future.

### Immunoconjugates

Immunoconjugates are macromolecular complexes comprised of a mAb that is connected to a cytotoxic agent with a linker that may be cleaved or not when reaching the targeted cell depending on which metabolic pathway the drug complex aims to take.^112^^,^^113^ Both non-human and human IgG (IgG1, IgG4, IgG2) are applicable in the therapeutic design, and the “drug missile” is commonly linked in the hinge region away from the antigen-binding or Fc effector functions by thiol-based conjugation. Such positioning is necessary, as disulfide bond cleavage may impede effective FcRn-dependent removal of immunoconjugates in the kidney.^114^ The antibodies loaded with cytotoxicity agents are called antibody-drug conjugates (ADCs). Generally, drug payloads are mainly comprised of microtubule-specific agents, DNA-cleavage agents, and alternative inhibitors against critical cell components including B-cell lymphoma extra large (Bcl-x_L_), RNA polymerase, and spliceosome.^115^^,^^116^ In general, 3−10 chemotherapeutic agents are latched onto an antibody. While traveling inside the bloodstream, ADC paratopes bind to targeted receptors present on the tumor cell surface, inducing a receptor-mediated endocytosis. As early endosomes form via clathrin-mediated endocytosis and acidify into late endosomes, a few ADCs bind to FcRn to be recycled. When lysosomes fuse with late endosomes, the linker is then broken, triggering the release of cytotoxic agents intracellularly.^117^^,^^118^ Appropriate selection of cytotoxic drugs and their linkers is critical for this pH-dependent cascading pathway to be successful. Other ADC-directed cell apoptosis pathways include ADCC and antibody-dependent cell-mediated phagocytosis (ADCP), both of which are induced by the IgG Fc region.

Belantamab mafodotin is one of the ADC therapeutics that has received an accelerated approval from the FDA in 2020 to treat patients with relapsed or refractory MM.^119^ Monomethyl auristatin F (mmAF), an anti-neoplastic agent that induces microtubule disruption, is conjugated to a humanized, afucosylated mAb that targets BCMA by a protease-resistant maleimidocaproyl linker.^120^ Afucosylation of antibodies leads to the extension of half-life and could allow less drug to be administered by increasing the affinity between the antibody and FcγRIII.^121^ BCMA is a member of TNF receptor superfamily, which is expressed in MM cells for cell survival and proliferation.^122^^,^^123^ By intracellularly delivering microtubule-disruptive agents inside BCMA-positive tumor cells, belantamab mafodotin strongly induces a pleiotropic anti-tumor activity against MM cells *in vitro* and *in vivo*.^124^ It also recruits NK cells and macrophages by improving binding affinity to FcγRIII so as to mediate ADCC and ADCP accordingly.^125^ In summary, belantamab mafodotin improved the progression-free survival in MM disease in a phase II clinical trial.^120^

Sacituzumab govitecan-hziy, or TRODELVY, is an ADC agent composed of anti-triophoblast cell-surface antigen 2 (Trop-2), hRS7 IgG1κ antibody conjugated to topoisomerase I inhibitor (SN-38), an active metabolite of irinotecan, with a cleavable CL2A linker.^126^ In April 22, 2020, it was approved by the FDA to be administered for adult patients against triple-negative breast cancer (TNBC), which is defined by the absence of estrogen receptors, HER2, and progesterone receptors.^127^ TNBC has been reported to overexpress Trop-2, a transmembrane glycoprotein transducer of calcium signal, for cancerous growth and apoptotic resistance.^128^^,^^129^ Although the direct correlation between the anti-cancer efficacy and Trop-2 inhibitory pathway of sacituzumab govitecan-haziy has not been confirmed, its receptor-mediated internalization and intracellular SN-38 delivery dramatically increased applicability of the topoisomerase I inhibitor, which had low tumor cell penetration and dose-limiting toxicity.^130^ Extracellular SN-38 activity was also reported due to the cleavability of the linker; bystander cancers that did not bind to ADC can also be killed by the therapeutic concentration of SN-38.^131^ In a phase I/II single-group trial, 108 pretreated patients with metastatic TNBC had received sacituzumab govitecan-hziy, where approximately 33.3% has responded for a median of 7.7 months after the drug was administered. 45.4% of patients received clinical benefits such that the overall survival of patients extended to 13.0 months, 5.5 months of which was post-treatment.^126^

Other ADCs that recently got approved for clinical application include trastuzumab deruxtecan and enfortumab vedotin. Trastuzumab deruxtecan is a humanized anti-HER2 mAb (trastuzumab) conjugated to the topoisomerase I inhibitor via a tetrapeptide linker that can be cleaved by cathepsin intracellularly.^132^ Loading nearly twice the amount of drug per antibody than transtuzumab emtansine, the conjugate exhibits an anti-tumor effect upon neighboring cancer cells due to the permeability of cytotoxic load through the cell membrane. The ADC is applicable in HER2^+^ cancers such as metastatic breast cancers (10%–20%) and advanced/metastatic gastric cancers (20%).^133^^,^^134^ In a phase II trial, out of 184 patients with HER2^+^ metastatic breast cancer and administered with trastuzumab deruxtecan, 60.9% responded for a median of 14.8 months. Although anemia, nausea, and interstitial lung disease were exhibited, the systemic damage was not critical due to short elimination half-life of the cytotoxic load.^133^ With a median of 16.4 months for progression-free survival, trastuzumab deruxtecan was approved by the FDA for anti-cancer administration on December 20, 2019.^135^

Enfortumab vedotin is a humanized conjugate between anti-Nectin 4 antibody and monomethyl auristatin E (mmAE), a microtubule-disrupting agent. Nectin-4, or poliovirus receptor-like 4, is commonly overexpressed in multiple organ-specific cancers for cell adhesion, proliferation, and growth.^136^ In a phase II trial (EV-201), the candidate drug was administered to patients with Nectin-4^+^ metastatic urothelial carcinoma; platinum-based chemotherapy and anti-PD-1/PD-L1 immunotherapies were given prior to ADC administration. Post-treatment adverse effects such as peripheral neuropathy (50%) and rash (48%) were present, but most were in grade 1 and manageable as these two mentioned; they were expected due to mmAE-associated toxicity and expression of Nectin 4 on dermal tissues, respectively.^137^ With 7.6 months of drug response as a median, enfortumab vedotin-ejfv became the first anti-Nectin 4 ADC agent to be approved by the FDA on December 18, 2019.^138^

### Chimeric antigen receptor (CAR) T cells

CAR T cells are allogenic or autologous CD4^+^/CD8^+^ T cells that are genetically engineering via CAR gene-containing viral infection (e.g., lentivirus) or CRISPR-Cas9 to express the transcripted CARs.^139^^,^^140^ Traditionally, T cell therapy was performed by isolating TILs from patients, expanding them with IL-2 *in vitro*, and infusing them back into the patient who has undergone lymphodepletion to lower the level of white blood cells. This was purposed to increase the number of T cells *in vivo* so as to enhance effector functions and immunological memories.^141^ CAR T cells were developed to directly fuse antigen-specific scFvs to intracellular signaling domains. CAR T cells secrete pro-inflammatory cytokines (e.g., IL-12) and chimeric cytokine receptor 4αβ (IL-4Rα-[IL-2/IL-15Rβ]) to activate a cascading signal pathway in response to IL-4.^142^^,^^143^ This subsequently results in either direct cell lysis by CD8^+^ cytotoxic T cells or immunological boost by CD4^+^ helper T cells. Although the targeted antigen can be expressed in normal tissues, resulting in inadvertent immune-related damage, accumulation of CAR T cells has shown promising tumor eradication in several tissues such as liver, lymph nodes, and bone marrows.^144^, ^145^, ^146^ CAR T cell therapy is sometimes combined with chemotherapy or low-dose irradiation regimens that would reduce levels of immuno-suppressive cells (e.g., T regulatory cells, myeloid cells) and increase the likelihood of CAR T cell survival. Brentjens et al.^147^^,^^148^ have experimentally discovered this controversial observation from patients with chemotherapy-refractory chronic lymphocytic leukemia (CLL) or relapsed B cell ALL.

Bi-specific anti-CD19/CD22 CAR T cell therapy has recently been spotlighted for its promising treatment of refractory diffuse large B cell lymphoma (DLBCL) compared to CD19 CAR T cell infusion, albeit the occurrence of CRS.^149^^,^^150^ Early stages of DLBCL are usually treated with four chemotherapeutic drugs (CHOP [cyclophosphamide, doxorubicin, vincristine, and prednisone]) or a combination of chemotherapy and immunotherapy (rituximab), whereas late stages undergo intense chemotherapy and allogeneic stem cell transplants.^151^^,^^152^ However, some patients cannot be cured with conventional regimens and thus have to resort to CAR T cell therapy instead. The two targets of anti-CD19/CD22 CAR T cells, CD19 and CD22, are commonly expressed on B cell lymphomas, whereas those receptors are not expressed on other cell types including hematopoietic stem cells.^150^^,^^153^ By targeting those two tumor-expressed receptors, the bi-specific immunotherapeutic agent resulted in a better outcome for DLBCL patients who expressed CD19 and CD22 heterogeneously than CAR T cells that targeted CD19 only. Dual CART T cells had also expanded approximately five times greater than did CD19-specific CAR T cell *in vivo*.^149^

Research and developments in CAR T cell therapy continue to lower CRS risk for broad application of the therapeutic agent. In 2020, Shi et al.^154^ reported the initial safety profile of CAR T cells against glypican-3 (GPC3) for treatment of hepatocellular carcinoma (HCC). Overexpressed GPC3 shows worse prognosis in HCC patients because GPC3 downregulates apoptosis by a dysfunctioning BCL2 associated X (Bax)/C-cell lymphoma 2 (Bcl-2)/cytochrome *c*/caspase-3 signaling pathway.^155^ After being administered with GPC3-specific CAR T cells, none of the patients has exhibited a grade 3 or 4 neurotoxicity. Only one patient had displayed symptoms of grade 5 CRS, and a patient had survived with a sustained stable disease for nearly 44.2 months. 10.5% of patients survived for 3 years, 42% for 1 year, and 50.3% for 6 months. Overall, the results from the phase I trial confirmed a good safety profile for GPC3-specific CAR T cells.^154^ Nevertheless, further research is necessary to minimize immune-related adverse effects (irAEs) of CAR T cells.

### Antibody mimetics

Despite their *in vivo* versability, antibodies are largely limited in various ways as follows: polyclonal preparation yields high variability, mAb production and chemical modifications are costly to ensure consistency, antibodies are vulnerable to heat and humidity, infusion triggers a size-dependent immune response, albeit the low tissue penetration.^156^, ^157^, ^158^ Antibody mimetics such as affibodies, adnectins, affimers, aptamers, designed ankyrin repeat proteins (DARPin), and knottin molecules are generated to feature properties that are desired, including pH stability, protease resistance, and absence of immunogenecity.^159^

Antibody mimetics are mainly constructed by either protein-directed evolution or CDR grafting guided by FR sequence homology. Directed evolution mimics the natural selection process, where proteins are constantly mutated and selected for optimization. A proper scaffold should be first selected based on how amenable it is to mutations and insertions. The focus is to ensure that any critical genetic mutations in those scaffolds would improve qualities of traditional antibodies without lowering binding affinity or specificity.^160^, ^161^, ^162^ Scaffolds are typically stable in temperature, non-glycosylated, and non-aggregable during protein expression. Once selected, the scaffold undergoes diversification by either error-prone polymerase chain reactions (PCR) or DNA shuffling; residues in ligand-binding sites are commonly targeted for site-directed or random mutagenesis.^159^ Once the library (nearly 10^12^ variants) is constructed *in silico*, and a few of mutated sequences are selected by a high-throughput screen or selection, the same process is iterated until a desired scaffold is generated. The resulting sequences can bind to their antigens of interest as high as femtomolar binding affinities. FR-guided CDR grafting constructs CDR-FR peptides by linking two CDRs with a cognate FR (from HC or LC) in between.^163^ Out of six CDR types that interact with antigens, the CDR3 loops in HC are commonly used due to their accessibility and sequence diversity. Either CDR1 or CDR2 are fused with CDRH3 via FR in a C- to N-terminal direction.^164^ Although antibody mimetics are generally limited due to their short half-life from Fc deletion, which cannot be fully recovered by artificial fusion or conjugation technologies such as PEGylation and PASylation, they still hold potentials as more stable and non-immunogenic alternatives to traditional IgG therapies.

Multiple of artificial constructs such as aptamers and DARPins have been recently developed and spotlighted as immunotherapeutic agents. Aptamers are small molecules consisting of single-stranded DNA or RNA (ssDNA or ssRNA) generated by an iterative process called systematic evolution of ligands by exponential enrichment (SELEX).^165^ A randomized library (10^12^−10^15^ sequences) consisting of (5′) constant-variable-constant (3′) structures is mixed with targets so that only target-binding sequences are isolated from the rest. After completing PCR amplification, the procured sequences repeat the same process usually for 9−15 times until the desired sequence is obtained.^166^ Several extensions to SELEX have been developed to shorten this iteration process (e.g., capillary electrophoretic SELEX, cell-SELEX, tailored-SELEX).^167^ Aptamers are highly noted for their low production costs, less restricting factors in subsequent modifications, low *in vivo* immunogenicity, and rapid tissue penetration. In direct therapy against tumor cells, the artificial ligands non-covalently bind to targeted receptors, triggering receptor-mediated endocytosis. Once escaping before lysosomal degradation, aptamers are assumed to trigger an intracellular signaling and induce a synthesis phase arrest of cells, inhibiting their growth and priming them for subsequent therapies.^168^^,^^169^ Cytarabine, a chemotherapeutic agent, is sometimes combined with aptamers for a precision lymphoma therapy.^170^ Aptamers are also applied for site-specific drug delivery, where they fuse nanomedicines into tumor cell membranes and enhance anti-tumor activity of the delivered cargo (e.g., small interfering RNA). Immunotherapeutic effects of NK cells can be enhanced by chemically modifying their surfaces with aptamers, which is similar to scFv-CAR T cell fusion.^171^^,^^172^ TLS11a and PD-L1 aptamers were fused to NK cells so as to enable both checkpoint blocking and selective NK-mediated tumor perforation and lysis. As a result, the modified NK cells demonstrated high secretion of granzyme B, perforins, and pro-inflammatory cytokines (IL-2, IFN-γ) without displaying *in vivo* toxicity.^173^ Aptamer-modified NK cells may be a safe alternative to CAR T cell therapy that has been previously reported for its severe CRS and acute anaphylaxis.

DARPins are 14−18 kDa ankyrin repeat proteins that are typically comprised of 33 amino acid residues. Each repeat contains a β-turn and two anti-parallel α helices after, and at most, 29 repeats can be generated in a monomer.^174^ Although having a large hydrophilic surface, DARPins shield their hydrophobic regions with C- and N-caps, which are integral for the folding process of the protein in *Escherichia coli*.^175^ Resembling to a curved solenoid, DARPins bind to their targets via 4−6 repeat domains. In designing a LoopDARPin library, which has a size greater than 10^10^ sequences, random amino acids (except cysteine, glycine, and proline) are inserted via the binding surfaces. The resulting proteins can achieve binding affinities to picomolar scales with a single round of ribosomal display, can maintain stability against heat (up to 90°C) and proteases, and can be mass produced in bacteria by up to 200 mg per liter.^174^^,^^176^ DARPins commonly target carcinogenesis-expressing molecules such as VEGF, HGF, HER2, and Kirsten rat sarcoma 2 viral oncogene homolog (KRAS).^177^, ^178^, ^179^, ^180^ Thanks to their structural rigidity and low molecular weights, DARPins can be multimerized or linked with other agents (e.g., radionuclide, nanoparticles, photosensitizer, oncolytic virus, CAR T cells) to enhance their functions such as immunosuppression, endocytosis-mediated toxin release, and Fc-mediated ADCC.^181^ In December 2019, Balakrishnan et al.^182^ generated multi-specific CAR T cells by replacing extracellular scFv with anti-EGFR, anti-EpCAM, and anti- HER2 DARPins. The tri-specific DARPin-CAR T cells showed comparable anti-tumor activity with traditional CAR T cells with a constant activation-induced T cell death rate when targeting single or multiple antigens, opening its possible applicability against heterogeneous tumors.^182^

## Discussion

Immunotherapeutic applications against cancer continue to show remarkable breakthroughs and promising clinical results in the 21^st^ century. Much of the successful performances of the antibody therapy stems from IC-mediated immune effects that include but are not limited to complement-dependent cytotoxicity, ADCC, and adaptive immunity, all of which are well established in various literature. Full-size antibodies are currently dominating the immunotherapy market as they require minimal modifications for production with tolerable pharmacological limitations in critical factors such as immunogenicity and tissue-penetration degree. However, as discussed in this review, multiple biochemical variations (e.g., BiTE, CAR T cells, antibody mimetics) have been extensively studied in pursuit of minimizing pharmacological limitations of full-size antibodies. It is true that current immunotherapy does exhibit considerable immune-related irAE such as hypophysitis induced by nivolumab administration; the baseline cost for the antibody treatment may not make it commonly affordable as of right now. Despite such limitations, considering the relatively recent introduction of immunotherapy under FDA approval compared to traditional anti-cancer treatments, it would be appropriate to assume the unexplored potential within the anti-cancer antibody as new interdisciplinary techniques such as convolutional neural networks are rapidly being integrated into the medicinal field for data-driven therapeutic optimization.
